# The Effect of Anesthesia on the Immune System in Colorectal Cancer Patients

**DOI:** 10.1155/2018/7940603

**Published:** 2018-04-01

**Authors:** Yangjie Dang, Xingxing Shi, William Xu, Mingzhang Zuo

**Affiliations:** ^1^Department of Anesthesiology, Xi'an Children's Hospital, Xi'an, Shaanxi 710032, China; ^2^Department of Anesthesiology, Carver College of Medicine, University of Iowa, Iowa City, IA 52246, USA; ^3^Department of Anesthesiology, Beijing Hospital, National Center of Gerontology, No. 1 Dahua Road, Dongdan, Beijing 100730, China

## Abstract

Colorectal cancer (CRC) is the key leading cause of high morbidity and mortality worldwide. Surgical excision is the most effective treatment for CRC. However, stress caused by surgery response can destroy the body's immunity and increase the likelihood of cancer dissemination and metastasis. Anesthesia is an effective way to control the stress response, and recent basic and clinical research has shown that anesthesia and related drugs can directly or indirectly affect the immune system of colorectal cancer patients during the perioperative period. Thus, these drugs may affect the prognosis of CRC surgery patients. This review is intended to summarize currently available data regarding the effects of anesthetics and related drugs on perioperative immune function and postoperative recurrence and metastasis in CRC patients. Determining the most suitable anesthesia for patients with CRC is of utmost importance.

## 1. Introduction

Colorectal cancer (CRC) is the third leading cause of death from cancer worldwide, accounting for approximately 135,430 new cases and 50,260 deaths in the United States in 2017 [1]. The interactions of the colon epithelium microbiome are considered to be important for the formation of colon cancer, and* Enterococcus faecalis* is thought to play an important role in the pathogenesis of CRC [2].

Anesthesia and related drugs can directly or indirectly affect the immune system of patients during the perioperative period and thus affect treatment and prognosis of CRC patients as surgery is currently the most effective treatment for CRC. The 2009 European Society of Anesthesiology (ESA) presented the new concept of “anticancer anesthesia technology” with the intention of identifying the most suitable anesthesia for patients with cancer. Exploring the effects of various anesthetic methods and their related drugs on the immune system of CDC patients continues to be of great significance.

## 2. Colorectal Cancer and Its Immunological Bases

Tumor generation requires the provision of nutrition in the surrounding microenvironment. Tumors grow and invade and infiltrate surrounding tissues and organs. The tumor microenvironment includes a large number of cells, including immune cells, endothelial cells, and interstitial cells, all of which are involved in the occurrence and development of tumors. Studies have shown that the immunological infiltration of CRC may be clinically related to those cells.

Immune response in the process of tumor development is not just a single factor, but it plays a multifaceted role affecting tumor initiation, growth, progression, and other processes. The immune system regulates and promotes cancer programs, a process known as “immunoediting.” There are three phases to this process: elimination, balance, and escape [3].

Although experimental evidence shows that inflammation can also promote the occurrence and development of tumors [2], the immune inflammatory response in colon carcinogenesis requires further study and is still under debate [4]. Some clinical data show that the immune response inhibits the tumor. However, other investigators have concluded that the opposite is true.

### 2.1. Immune Cells in Colorectal Cancer

Different immune cells play differing roles in the process of cancer: some cells affect its pathogenesis while others contribute to its recurrence.

*Macrophages*. Macrophages in CRC will appear as infiltration. Known as tumor-infiltrating macrophages, they are important contributors to tumors infiltrating the surrounding tissue. They are derived from peripheral blood mononuclear cells. According to the different functions and characteristics of tumor-infiltrating macrophages, they can be divided into two different subtypes: M1 and M2 [5]. Some cytokines, such as LPS and TNF-*α*, promote the conversion of classic activated macrophages to the M1 type. The remaining types are classified as M2. M1 is involved in the TH1-type immune response that can kill foreign pathogens and endogenous tumor cells. Glucocorticoids and IL-10 can induce the transformation of tumor-infiltrating macrophages to M2, which mainly causes TH2-type immune response and promotes the occurrence and development of tumors. Both types are involved in the immune response of colon cancer and can be transformed into each other under certain circumstances.

In short, both M1 and M2 participate in the occurrence and outcome of the tumor, and the conversion between the two determines the outcome and prognosis of tumors [6, 7]. Some studies have shown that macrophage infiltration in CRC seems to prefigure a better prognosis [8], since macrophage infiltration may be an important means of fighting against cancer via the regulation of endogenous mechanisms.

*NK Cells*. Natural killer (NK) cells are immune cells that maintain the defense function of the body. They are involved in antitumor and antiviral infection and immune regulation processes. Both murine [9, 10] and human [11, 12] models have shown that NK cells contribute to preventing tumor and controlling the effects of tumor growth and dissemination. In CRC, it has been reported that extensive infiltration of NK cells in a wide range of tumors can give rise to a better prognosis [13]. Although these studies suggest that NK cells play a beneficial role in tumor control, their specific mechanism of action remains unclear [14]. Therefore, as an important effector cell in innate immunity, NK cells have significant antitumor function and have the potential for positive applications and clinical significance in tumor immunotherapy.

*T-Regulatory Cells (Tregs)*. Tregs, CD25 + regulatory T-cells, can not only prevent human autoimmune diseases, but also help protect against microbial infection and protect the fetus. There are indications that Tregs can indeed prevent human autoimmune diseases [15]. On the one hand, Tregs may control excessive immune response, but on the other hand, such control may also weaken the immune system's effectiveness in eliminating invaders. The most direct application for Tregs is in treating autoimmune diseases by increasing their activity. In addition, reducing the number of Tregs can also reduce tumor-associated immune responses. There is evidence that immune cells constantly monitor the molecular abnormalities that occur when cells become cancerous. To some extent, Tregs interfere with this monitoring and may help to root and grow malignant tumors [16]. On the basis of these studies, it was also found that tumor-associated T-cell-related immune responses are detected in tumor patients [17, 18]. Numerous studies have shown that T-cells infiltrate tumor tissue in patients who are less immunosuppressed. This is considered to be a good prognostic marker [19, 20]. Therefore, modulating the immune response of tumor patients can be an important way to improve the prognosis of patients, but further studies are needed.

### 2.2. Immune System and Colorectal Cancer

The innate immune response is a nonspecific immune defense already available at birth. This inherent immune response has a wide range of effects, including speed, efficiency, and stability. Innate immunity provides a nonselective rejection to a foreign body's entry into the body of antigens, a protective clearance function. The innate immune system is activated through the recognition of pathogen-associated molecular patterns (PAMPs) by pattern recognition receptors (PRRs) which, in turn, produce a series of cytokines such as interleukins (ILs) and tumor necrosis factor (TNF). The innate immune response initiated by PRRs is important in the shaping of tumor immune microenvironment and tumorigenesis. PRRs are widely expressed in various innate immune cells of the host, such as dendritic cells and mononuclear macrophage NK cells, after which a series of immune responses are initiated. Among various PRRs, Toll-like receptors (TLRs) have attracted much attention and are involved in tumorigenesis through the recognition of the inflammation caused by PAMP. Among these receptors, the inflammasomes formed by the involvement of Nod-like receptors (NLRs) have also received much recent attention, as they have been shown to regulate the immune response and thus to inhibit tumorigenesis [21, 22].

Rakoff-Nahoum et al. [23] studied the dextran sulfate sodium- (DSS-) induced susceptibility to colitis and receptor-interacting protein 2 (RIP2) in TLR4, TLR2, and myeloid differentiation factor (MyD88) knockout mice in 2004. Compared with wild-type (WT) mice, they demonstrated that the MyD88-dependent axis is beneficial. MyD88 is a key linker molecule in the TLR-signaling pathway and plays an important role in the transmission of upstream information and disease development. It is therefore an important mediator of many molecular cascade reactions.

In addition, interleukin-17 (IL-17) is an early promoter of T-cell-induced inflammatory responses and can amplify the inflammatory response by promoting the release of proinflammatory cytokines. The main effector of T-helper interleukin-17-producing cells (Th17 cells) is IL-17. Th17 cells are capable of secreting IL-6 and tumor necrosis factor-*α* (TNF-*α*), among others. Subsequently, one study [24] has demonstrated that IL-17 plays a role in the initiation of tumors in IL-17 knockout mice.

At present, the main treatment for CRC is surgery. Therefore, anesthetic management plays a vital role in the prognosis of CRC in the perioperative period.

## 3. The Effect of Anesthesia on the Immune System in Colorectal Cancer Patients

The immune system mainly includes two subsystems: nonspecific immunity and specific immunity. The effect of anesthesia on the immunization of colorectal cancer patients mainly includes the following. (1) Anesthesia affects the number and activity of immune cells: presently, the effects of anesthesia on immunity have been studied on NK cells, B-lymphocytes, T-lymphocytes, macrophages, leukocytes, and erythrocytes. These cells are involved in both nonspecific and specific immune functions. (2) Anesthesia affects the secretion of cytokines. Proinflammatory factors mainly include tumor necrosis factor-a (TNF-*α*), IL-1, IL-6, and IL-8. The anti-inflammatory factors mainly include IL-10. The levels of interferon-7 (IFN-7), interferon (IFN), TNF-*α*, and soluble interleukin-2 receptor (sIL-2R) are increased by general anesthesia. (3) Anesthesia affects the biological behavior of tumor cells. The proliferation, migration, and apoptosis are important biological characteristics of tumor cells, which are closely related to tumor growth and metastasis.

### 3.1. The Effect of Different Anesthesia Methods on Immunization in Colorectal Cancer Patients

A number of scholars have found that, compared to simple general anesthesia, general anesthesia combined with epidural anesthesia or simple spinal anesthesia can reduce the postoperative immunosuppression to varying degrees [25]. Studies on the effects of different anesthesia methods on the long-term survival rate of patients after colon cancer resection suggested that the combined application of epidurals can improve the survival rate of patients without metastasis to 1.46 years postoperatively, but there was no significant difference between the two groups for patients with metastasized tumors [26]. These studies have shown that, compared to general anesthesia, epidural anesthesia, paravertebral block, and other types of regional anesthesia can reduce the inhibition of immune function and improve the prognosis of patients with cancer.

Surgery can cause sympathetic adrenal medulla and hypothalamus-pituitary-adrenal cortical axis excitement based stress response by a variety of factors (pain, blood loss, low temperature, and psychological factors). Many factors produced in the stress response, such as glucocorticoids, catecholamines, and prostaglandin E2 (PGE2), can have an adverse effect on immune function, resulting in immune function inhibition in the perioperative period. Studies have shown that the inhibition of immune function by stress response is caused by inhibition of natural killer cell (NK cell) function. These cells are nonspecific components of cellular immunity. Their effect is not tumor-specific and the major histocompatibility antigen system (MHC) restriction plays an important role in early antitumor immunity. The effect of the stress response on the duration of NK cell activity inhibition is similar to the period of enhanced tumor metastasis [27]. The effect of the stress response on tumor metastasis is achieved by inhibiting the activity of NK cells. Epidural anesthesia can block the pain-conduction pathways, non-pain-conduction pathways, and sympathetic nerve fibers by blocking the spinal nerve root. However, opioid drugs commonly used in general anesthesia only block the pain-conduction pathway [28], indicating that epidural anesthesia can preserve NK cell function. Compared to general anesthesia, epidural anesthesia can better prevent nociceptive irritation transmission to the central nervous system and reduce the body's stress response, which may be beneficial for the prognosis of patients with cancer.

CD4+ T-cells first differentiate into T-helper 0 (TH0) cells after antigen stimulation and continue to differentiate into TH1 or TH2 cells. TH1 or TH2 subgroups secrete different cytokines: TH1 cells mainly secrete interferon-1 (IFN-1), which promotes cellular immunity, while TH2 cells secrete interleukin-4 (IL-4) and are associated with humoral immunity. These cytokines can also promote their own growth and inhibit the growth of other subpopulations. The proportion of IFN-*γ* IL-4* in vivo* determines the ratio of TH1/TH2 which, in turn, determines the dominant* in vivo* immune response. Cellular immunity is important in controlling the immune response of the tumor, and thus a reduced TH1/TH2 ratio should be avoided in order to maintain the cell's immune response. Epidural anesthesia, compared to general anesthesia, is able to better preserve the TH1/TH2 ratio and thus can better preserve cellular immune function. Mrakovcic-Sutic et al. found that, in rectal cancer patients with postoperative analgesia, there was a lower inhibitory effect on NK cells than in patients in an intravenous analgesia group [29]. Animal experiments suggested that epidural anesthesia can increase the survival rate of colorectal cancer patients after surgery. The number of lymphocytes, NK cell activity, TH1/TH2 ratio, and tumor metastasis were significantly higher than those of general anesthesia group after spinal anesthesia, indicating that specific regional anesthesia could preserve the function of immune cells. Therefore, general anesthesia combined with epidural anesthesia or simple spinal anesthesia is better than simple general anesthesia in reducing surgery-related immune inhibition by reducing the stress response.

The protective mechanism of local anesthesia technology on immune function is not yet clear; however, there are three possible causes: (1) local anesthesia technology, by blocking the neurotransmitter incoming to the central nervous system, can significantly reduce the pain and surgical stress response; (2) the application of local anesthesia technology can reduce the dosage of opioids; and (3) general anesthesia combined with local anesthesia techniques can reduce the total use of general anesthetic drugs. Epidural anesthesia combined with general anesthesia can reduce the amount of general anesthetics and analgesics, and sustained epidural analgesia can also further reduce the use of opioids after surgery, which may be one of the reasons for reducing the immunosuppressive effects.

However, some scholars still believe that local anesthesia technology cannot protect the immune function and reduce tumor recurrence and metastasis. Gottschalk et al. analyzed the data of 669 colorectal cancer patients undergoing radical surgery and found that the application of epidural analgesia during the perioperative period and a reduced recurrence rate of tumor after surgery are not necessarily linked [30]. The meta-analysis of Conrick-Martin et al. suggests that there was no significant difference between general anesthesia and spinal anesthesia on NK cell function after surgery [31]. The existing clinical research sample size is small, and retrospective studies have inherent bias and other deficiencies. In the future, more multidimensional, prospective, randomized clinical studies will be needed to further explore the linkages and mechanisms between anesthetics and the prognosis of malignant tumors in order to provide safer anesthesia for patients with cancer. For future study, retrospective studies will also require forward-looking large samples.

### 3.2. The Impact of Anesthesia-Related Drugs on Immunization in Colorectal Cancer Patients

#### 3.2.1. Intravenous Anesthetics

Intravenous anesthesia can maintain a safe, constant concentration of drug treatment during surgery and can reduce the stimulation of surgical trauma. Intravenous anesthesia can also reduce the intraoperative inflammatory response albeit affecting the patient's immune system function. Immune system disorders or inhibition during the perioperative period can cause postoperative complications, especially in cancer patients. Immunosuppression after surgery can accelerate the spread of residual cancer cells and promote a new transfer.

Common opinion is that propofol does not inhibit the immune function. Inada et al. [32] found that propofol produces an antitumor effect by inhibiting cyclooxygenase-2 (COX-2) and prostaglandin E2 (PGE2). Propofol is superior to inhaled anesthetics in postoperative immunoprotection. Propofol can increase the activity of NK cells, inhibit COX-2, prevent PGE2 generation, and protect the immune system function. It can also have a direct biological effect on the tumor. A series of cell culture experiments show that propofol at concentrations commonly used does not inhibit phytohemagglutinin- (PHA-) induced lymphocyte proliferation and does not significantly increase lymphocyte apoptosis. Propofol inhibits immune cells only at concentrations well above clinical concentrations [33–36].

Ketamine, thiophanate, and etomidate increase the chance of tumor retention and metastasis because of the reduced NK cell activity, reduced T-helper cells, and increased T-inhibiting cell viability. Ketamine inhibits NK cell activity due to the activation of alpha and beta adrenergic receptors [37].

Benzodiazepines are one of the most commonly used sedatives in intensive care patients [38]. Midazolam can inhibit the production of IL-2 and IL-8 to produce an immunosuppression effect [39]. Midazolam can inhibit the transcription activity of lipopolysaccharide-induced nuclear factor KB, the activity of TNF-*α*, the phosphorylation of p38 mitogen kinase, and the formation of peroxides, which can be blocked by peripheral benzodiazepine agonist PKII 195. Midazolam can inhibit neutrophil adhesion and tropism by inhibiting the level of IL-8, thereby reducing immune function [40]. Rapid administration of diazepam will produce a proinflammatory response and improve neutrophil function, but slow and sustained (60 days or longer) administration will inhibit the multinuclear leukocyte function and lead to immune response inhibition [41]. These results show that benzodiazepines have a significant inhibitory effect on innate immunity, but more research is needed on adaptive immunity.

The *α*2-agonists clonidine and dexmedetomidine can significantly increase tumor cell growth, but the *α*2-receptor antagonist yohimbine can reverse this effect [42].

#### 3.2.2. Inhaled Anesthetics

Inhaled anesthetics include sevoflurane, halothane, isoflurane, and desflurane. The inhibitory effect of inhaled anesthetics on the immune function has been confirmed by many studies. Guptill et al. found that intravenous anesthesia with propofol and remifentanil is less immunosuppressive than inhaled anesthesia [43]. Inhaled anesthetics have greater immune cell inhibition than intravenous anesthetic drugs. Inhaled anesthetics can produce higher levels of plasma catecholamines and glucocorticoids to inhibit the release of IFN of NK cells in animal models and reduce the number of NK cells, the production of Th cytokines, and the expression of Foxp_3_ mRNA in the human body [44].

Some studies have shown that halothane inhibits NK cell activity in a dose-dependent manner.* In vitro* experiments show that an increase in halothane concentration is accompanied with a significant reduction of NK cell activity. With an increase in halothane concentration, NK cell activity was significantly reduced in* in vitro* experiments [45].

Other studies have reported that sevoflurane can reduce the invasive ability of colorectal cancer [46].

Fleischmann et al. conducted a follow-up study with 204 colorectal cancer surgery patients and found that the use of nitrous oxide (N_2_O) anesthesia does not increase the risk of recurrence of postoperative rectal cancer. However, the mechanism by which inhaled anesthetics affect the immune system remains unknown [47].

#### 3.2.3. Opioid Analgesics

Acute pain is the main cause of the activation of the hypothalamic-pituitary-adrenal axis (HPA axis), the activation of which can cause immunosuppression, decreased NK cell activity, and Th cell imbalance. Opioid is the main analgesic in anesthesia, and now the study found that opioids and tumors have a complex association. The mechanism is unknown and even, to some extent, contradictory.

Opioid itself can directly affect the proliferation, apoptosis, and survival of normal cells and tumor cells. Opioid analgesics have the effect of inhibiting cell and humoral immunity. In isolated experiments, morphine, a commonly used opioid, can inhibit the formation of peroxides and the expression of cytokines. Morphine has also been found to inhibit the function of macrophages and NK cell activity [48].

Morphine regulates tumor cell invasion throughout adhesion, extracellular matrix degradation, and cell migration. An opioid represented by morphine can regulate angiogenesis, promote endothelial cell proliferation and migration, promote angiogenesis, increase vascular permeability, and promote the proliferation of tumor cells in the blood vessels [49].

Another commonly used opioid, fentanyl, exhibited dose-dependent cytotoxicity against NK cells. Forget et al. found that fentanyl was able to inhibit the activity of NK cells in mice [48]. The effect of different doses of fentanyl on human immune function was observed. It was found that the activity of NK cells was reduced in patients with both low and high doses of fentanyl at 24 hours after operation. However, the activity of NK cells in the low-dose group returned to the preoperative level after 48 hours, while NK cell activity in the high-dose group remained low, indicating that the inhibitory effect of fentanyl on NK cells was time- and dose-dependent [49]. However, some studies have suggested that opioids can enhance the immune function by enhancing NK cell activity and the T-cell-mediated immune response. One possible mechanism involves morphine, which can activate the *μ* receptor and inhibit the release of NF-KB and NO [50, 51].

Sufentanil is a potent opioid analgesic, but it is unclear if it negatively affects the immune function.

Tramadol is a class of weak opioid receptor agonists that play an important role in the current clinical “multimodal analgesia” concept. They exhibit protective effects on cellular immunity in some studies.

#### 3.2.4. Nonsteroidal Anti-Inflammatory Analgesics

Acute and chronic inflammation significantly increases the expression of cyclooxygenase (COX), causing increased prostaglandins, pain, and tumor metastasis rate. Nonsteroidal anti-inflammatory drugs (NSAIDs) have antipyretic, analgesic, anti-inflammatory, and antirheumatic effects. They work by reducing the biosynthesis of prostaglandin (PG) in the local tissue by inhibiting the activity of COX* in vivo*. Because of the strong analgesic effect of nonsteroidal anti-inflammatory drugs, researchers have been concerned in recent years about whether NSAIDs provide immune protection during the perioperative period and about whether or not they relieve the recurrence of tumor metastasis. NSAIDs can improve the immune function of patients and inhibit the recurrence of tumor metastasis. It has been suggested that NSAIDs can be used to reduce pain, reduce opioid dosage, and balance side effects of opioids in patients with radical neoplasm surgery. Animal models suggest that COX inhibitors can prevent tumor growth and metastasis by inhibiting apoptosis and reducing angiogenetic factors and tumor microvessel density. Epidemiological studies have shown that prolonged use of COX inhibitors can reduce the risk of cancer. The use of selective COX inhibitor celecoxib daily can reduce the risk of colorectal cancer by 69% [52]. While encouraging, this conclusion requires further study. In addition to inhibiting the synthesis of PG, NSAIDs also inhibit the release of bradykinin during inflammation, alter lymphocyte responses, and reduce the migration and phagocytosis of granulocytes and monocytes to regulate the immune response [53]. NSAIDs can inhibit the immunosuppression caused by trauma, pain, anesthesia, opioid use, surgery, and so forth to varying degrees [54–56].

We could use COX inhibitors to inhibit PG synthesis in order to slow down tumor progression. Anesthesia and analgesia are associated with the entire perioperative period, although they cannot resolve the issues of tumor spread and tumor residue. However, reducing NK cell activity inhibition and maintaining Th cell balance can improve the immunosuppression caused by surgical stress induced by some anesthesia intervention.

#### 3.2.5. *β*-Receptor Blockers

The use of beta-blockers can reduce the risk of tumor metastasis by inhibiting the release of catecholamines and the activity of signal transducer and activator of transcription-3 (STAT-3), resulting in reduced NK cell activity inhibition and angiogenesis [57].

#### 3.2.6. Local Anesthetics

Most studies suggest that local anesthetics have antitumor effects and are suitable for anesthesia in patients with cancer. Martinsson found that the clinical concentration of ropivacaine can inhibit colon cancer cell proliferation in dose-dependent* in vitro* experiments [58]. Lucchinetti et al. found that bupivacaine can inhibit mesenchymal stem cell proliferation and negatively regulate tumor formation, metastasis, and cell differentiation [59].

### 3.3. The Effects of Other Anesthesia Interventions

#### 3.3.1. Heat Preservation

As a stress response, hypothermia activates the sympathetic system and increases glucocorticoid release. A mild temperature (35.5°C) has some effect on cellular immunity, but a moderately low temperature (30°C) will directly inhibit NK cell activity and reduce resistance to tumor metastasis [60]. Therefore, the use of a warm blanket, hot air, infusion of liquid heating, and other measures ensures that patients maintain the appropriate body temperature perioperatively, which can offer protection for the immune function in cancer patients after surgery.

#### 3.3.2. Blood Transfusion

While blood transfusion is an important tool for anesthesiologists during and after operation, perioperative blood use may influence cancer recurrence depending on the patient's nutritional health, anemic status, tumor type, stage and degree of resectability, blood loss, anesthesia type, stress level, and postoperative complications [61–67]. It has been reported that perioperative blood product transfusions, including packed red blood cells, platelets, and fresh frozen plasma, are linked to an increased risk of cancer recurrence in CRC patients [68–72]. Strict control of transfusions should be suggested to avoid unnecessary and excessive blood transfusion.

#### 3.3.3. Mood

Anxiety in patients with cancer is associated with postoperative immunosuppressive levels, and the anesthesiologist can help to relieve the patient's anxiety about surgery and disease by preoperative conversation and medication [73].

## Additional Points

*Summary*. Perioperative factors such as surgical trauma, volatile anesthetics, opioid use, physiological stress, hyperglycemia, hypothermia, blood products transfusion, and mood can cause a significant TH1/TH2 imbalance between the antitumor and protumor environments in the human body and can have a profound effect on the initiation and progression of colon carcinogenesis, colon cancer metastasis, recurrence, response to standard antitumor therapy, and the final clinical outcome. In brief, regional anesthesia is significantly better than general anesthesia in the immune index and in later tumor recurrence and metastasis. General anesthesia combined with regional anesthesia is better than single-use general anesthesia. Most inhaled anesthetics, opioids, local anesthetics, and other intravenous anesthetics can reduce immunity to a certain extent, which sometimes leads to an increased recurrence of malignant tumors. However, tramadol, selective nonsteroidal anti-inflammatory analgesics, and propofol have protective effects on the immune function of the body and can reduce the recurrence and metastasis of the tumor. Therefore, it is important to make a careful anesthesia plan and to select appropriate narcotic drugs for patients with malignant tumors, since these decisions will have a crucial impact on the therapeutic effect and prognosis. In addition, red blood cells, platelets, and FFP transfusion during the perioperative period also directly cause immunosuppression and increase the risk of cancer recurrence in colorectal patients. Hypothermia, hyperglycemia, and even a patient's mood contribute to changes in immunity and clinical outcomes in colorectal cancer perioperatively. Existing clinical research sample sizes are small, and retrospective studies have inherent bias and other deficiencies. In the future, more basic research and large-scale, prospective, randomized clinical studies will be needed to further explore the linkage and mechanisms between anesthetic factors and the prognosis of malignant tumors to provide safer anesthesia for cancer patients.
